# The Collection and Shipping of Pathological Specimens

**Published:** 1916-09

**Authors:** G. M. Graham

**Affiliations:** State Bacteriologist


### The Collection and Shipping of Pathological Specimens.

BY DR. G. M. GRAHAM, STATE BACTERIOLOGIST.

Within the last few years invaluable advances have been made in every branch of medicine, and permanent theories have come to replace the doubt and instability of past years. The trend of medical investigation at the present time is toward the most sensible and practical idea, prevention of disease, and education, sanitation and vaccination are now essential weapons against disease agents.

The clincal laboratory has contributed much to this cause, and renowned laboratory men, like Pasteur, Koch, and Wright, have been leaders in prevention of disease. They have taught the medical profession that attention to the minute details is essential to success, as only by the utmost carefulness in the laboratory is the pathologist able to achieve results. He uses measurements in his work which figure down to a fraction of a mm., a degree of temperature, and of seconds. Now, if these details are so essential in the laboratory in working with specimens, they are almost as essential in the collection of specimens to be sent to the laboratory.

Every laboratory man is worried by the fact that many specimens sent to him are not collected or shipped properly and, even though he is able to examine them, he is unable to interpret correctly his results. To be of any service to the practitioner and to give intelligent and definite reports, he must receive specimens which have been properly prepared. Most every day we get material which we are unable to examine—for example, sputum which has not been well sealed, and has leaked out and saturated the whole container and wrapping,—a piece of paper with a thin smear of feces to be examined for hookworm, or a specimen of boiled water for bacteriological examination.

First, specimens should all be plainly labeled—not on the outside wrapping, but on the container itself. We get many packages which have no means of identification except the post mark. Secondly, every one should be accompanied by a letter of explanation and history. Of course a history is not necessary in every case, but in some it is absolutely essential. Many physicians believe it best not to send a history, as it much influences the pathologist in his report. Of course it might influence some, but not if he is honest and competent. The pathologist wants the history in order to be able to better read his results, and give an intelligent report. For instance, it is a well known fact that 100 c.c. of alcohol taken by the patient the day blood is withdrawn for a Wasserman will reverse the test, or, in other words, make a positive Wasserman negative. Anti-syphilitic treatment will do the same thing. Now if we do the test and get the negative result, not knowing this history, our report is not only worthless, but is misleading. In a case like this, and in many other instances, a history is absolutely necessary, and for this reason it is always best to send one with the specimen.

Now, in regard to the proper collection of blood specimens, we will take up the Wasserman.

With every specimen of blood sent in for the Wasserman the laboratory man deserves a history. Tell him how long since the initial lesion, if any, the amount of treatment, and the prevailing symptoms; also try to exclude yaws, leprosy, T. B., malaria, scarlet fever and cancer, as in some cases these diseases give a positive Wasserman. As said above, do not withdraw blood from a patient who is drinking, or has been the day before. As a general rule it is also worthless to test a patient's blood who has had treatment in the preceding six weeks, as the treatment will reverse the test, and the patient may still have syphilis. In collecting the blood we advise the use of a Wright's capsule, which can easily be made with a thin glass tube and a flame. Prick deeply the end of the flexor surface of the finger, and milk the finger toward the end. As each drop collects apply the curved part of the tube to the drop, and capillary attraction will fill the tube. The trick of the method consists in applying the tube only when each large drop collects. After the tube is two-thirds full, gently heat the straight end and seal it, then when it cools, shake the blood down into the straight end as you would a clinical thermometer. The curved end can then be sealed. In a patient in whom you suspect syphilis of the central nervous system, the cerebro-spinal fluid will give positive results sometimes when the Wasserman in the blood is negative. This fluid can be sent in a sterile test tube tightly sealed.

In collecting blood for the Widal reaction, use the above method; that is, in a Wright capsule. Some laboratory men advise sending in a large drop of dried blood on a slide, but, in my experience this method has not proved satisfactory, as the dilutions are not accurate. It is practically useless to have the Widal made in the first week of a suspected case of typhoid, as it is rarely positive at this time. However, blood cultures give a large per cent of positive results during the earlier stage.

It is practically impossible for the general practitioner to make a blood culture, as he has not the necessary media, etc. The method we use is to withdraw 10-20 c.c. of blood from a prominent vein on the forearm, and inoculate about 75 c.c. of bouillon; let it incubate for twenty-four hours and examine. Of course this procedure varies with the organism we suspect.

Smears sent to be examined for malaria should be smeared thin, and collected, if possible, just before the patient has a paroxysm. At least two smears should be made, and separated by the usual method.

*Anthrax.*—The blood taken from animals recently dead of charbon will always show many germs (anthrax bacilli). From the suspicioned animal secure the blood by making a small wound in the skin and even into one of the superficial veins, being careful that none of the exuding blood falls on the ground or gets on the one securing the specimen. Use a small medicine dropper, having been previously sterilized by boiling, and draw the glass tube about one-half full of the suspected blood. Inject this blood into a small glass bottle, which has been previously sterilized by boiling for thirty minutes, and apply the stopper tightly. Prepare it for mailing by putting it in a case of some kind and send to the laboratory. Accompanying the specimen should be the name and postoffice address of the sender and a request for an examination for charbon germs (anthrax bacilli). Send to the State Board of Health, Austin, Texas, and upon its receipt an examination will be made and a report of the findings promptly sent, free of all cost. The germs of charbon are not often found in blood of animals still living, even though they may be infected with this disease; therefore, negative reports on blood taken from living animals are not always conclusive evidence.

The above method is preferable for sending specimens to the laboratory, but, in case it is impracticable, you may prepare the blood as follows:

From the suspicioned animal secure a drop of blood by making a small wound in the skin and even into one of the superficial veins, being careful that none of the exuding blood falls on the ground. Place a drop of this blood on a piece of clean, clear glass (one inch wide and two inches long); smear the drop of blood out into a thin film and let this film of blood dry in the sun. When the film has dried, carefully wrap the glass slide in a piece of paper.

Blood counts, estimation of Hb, etc., are usually taken directly by the pathologist, and I will not worry you with the technique.

All pathologists are worried by the way they receive the specimens of sputum. Aside from the unpleasantness and danger of handling an improperly prepared sputum specimen, we cannot report definitely. Sputums for examination should be collected in a wide mouth bottle, tightly sealed, wiped off well on the outside with some disinfectant, packed well in cotton and shipped to the laboratory at once. Try to collect the expectoration from deep down in the lungs, not mucus from the nose and throat. An early morning specimen is best, as we get this secretion from the whole night. And, another point,—one negative examination is not conclusive evidence that the sputum does not contain T. B. bacilli; only repeated examinations. I have in mind one case which had all the clinical symptoms of T. B.—yet only after eight examinations did we find the tubercle bacilli.

Feces to be examined for intestinal parasites should be collected from a normal action. Do not give salts, calomel, etc., for these dilute the action and make the examination more difficult. For a test, send in a small portion of the normal bowel action (say, about the size of a marble) and with it the name, age, symptoms, etc., of the patient. In examining for amoebae, the stool must be kept warm, as only by their motility are we able to recognize them definitely.

Throat cultures from suspected diphtheretic patients should be made from the membrane with a sterile swab, and inoculated immediately on Loeffler's blood serum, then sent at once to the laboratory. A swab made with unsterile cotton is worthless. Often we receive swabs of cotton which have been sent hundreds of miles; by the time they reach us all organisms are dried out and dead, and we are unable to get a growth. (If the patient shows clinical symptoms of diphtheria, give the antitoxin without waiting for a report.)

In sending urine for an ordinary chemical and microscopical examination, just collect in a bottle and ship immediately. If a vaccine is desired, the urine should be collected under sterile conditions by catheter, and shipped in a sterile bottle. In examinations for T. B., a twenty-four-hour specimen should be collectced under sterile precautions, for then the pathologist has a greater chance of finding the bacilli, which is always difficult.

In collecting water for bacteriological examination, special precaution must be taken to prevent risk of contamination. Small white glass bottles with glass stoppers, having a capacity of at least a pint, should be used. Both bottle and stopper should be well cleansed, and then boiled for at least fifteen minutes. After removing from the boiling water, the bottle should be well drained and the stopper inserted.

The water collected in such containers can be used for determining the number of bacteria and for the examination for particular organisms, such as B. Coli. If a quantitative examination is desired, the samples should be packed in ice and shipped immediately to the laboratory.

The following instructions for collecting specimens may be useful:

(1) Use white glass bottles, with glass stoppers.

(2) Rinse bottle three times with water to be examined before filling and corking.

(3) In collecting from a pipe, turn tap on and allow water to run at least two minutes. It is also a good idea to flame the nozzle well with burning cotton before collecting.

(4) In collecting from a stream, lake, spring or well, the stopper should be inserted in the bottle and whole bottle placed well under the surface of the water, the stopper removed, and the bottle filled. This prevents collection of surface water.

(5) When collecting samples, hold the bottle with the hand as far away from its neck as possible.

(6) Secure the stopper by clean linen or cord.

(7) Label the bottle, stating the source, date, time of collection, name of sender.

The following is an extract from the United States postal laws and regulations governing the shipment of pathological specimens through the mail:

Section 473, United States Postal Laws and Regulations:

1. Specimens of diseased tissue may be admitted to the mail for transmission to United States municipal, or other laboratories in possession of permits only when enclosed in mailing cases constructed in accordance with this regulation, provided that bacteriologic or pathologic specimens of plague and cholera shall under no circumstances be admitted to the mail.

11. Liquid cultures or cultures of micro-organisms in media that are fluid at the ordinary temperature (below 45° C. or 113° F.) are unavailable. Such specimens may be sent in media that remains solid at ordinary temperature.

4. A. Specimens of T. B. sputum (whether disinfected with carbolic acid or not disinfected) shall be transmitted in a solid glass vial with a mouth not less than one inch in diameter and a capacity of not more than two ounces, closed by a cork stopper or by a metalic screw top protected by rubber or felt washer. Specimens of diphtheria, typhoid, or other infectious or communicable diseases or diseased tissues shall be placed in a test tube made of tough glass not over three-fourths of an inch in diameter, not over seven and one-half inches in length, closed with a stopper of rubber or cotton and sealed with paraffin or covered with a tightly fitting rubber cap.

B. The glass vial or test tube shall then be placed in a cylindrical tin box with soldered joints closed by a metal screw cover, with a rubber or felt washer. The vial or test tube in this tin box shall be completely and evenly surrounded by absorbent cotton closely packed.

C. The tin box with its contents must then be closed in a closely fitting metal, wooden or papier-mache block or tube at least three-sixteenths of an inch thick in its thinnest part, of sufficient strength to resist rough handling and support the weight of the mails piled in bags. This last tube shall be tightly closed with a screw top cover with sufficient screw threads to require at least one and one-half full turns before it will come off, and fitted with a felt or rubber washer.

5. Specimens of blood dried on glass microscopic slides for the diagnosis of malaria or typhoid fever may be sent in any strong mailing case which is not liable to breakage or loss of the specimen in transit.

6. Upon the outside of diseased tissues admitted to the mails shall be written or printed the words, "Specimen for bacteriological examination. This package to be pouched with the letter mail."

---

Dr. B. F. Stout, of San Antonio, is in the North doing post-graduate work.

---

The National Association for the Study and Prevention of Tuberculosis announces that December 3rd to 10th has been designated as "Tuberculosis Week." All kinds of suitable literature have been prepared for those interested in this movement, and can be obtained at a reasonable price by application to the National Association, 105 East Twenty-second Street, New York.

